# Characterization of Behaviour and Remote Degeneration Following Thalamic Stroke in the Rat

**DOI:** 10.3390/ijms160613921

**Published:** 2015-06-17

**Authors:** Nina Weishaupt, Patricia Riccio, Taylor Dobbs, Vladimir C. Hachinski, Shawn N. Whitehead

**Affiliations:** 1 Department of Anatomy and Cell Biology, Schulich School of Medicine and Dentistry, University of Western Ontario, London, ON N6A 5C1, Canada; E-Mails: nweishau@uwo.ca (N.W.); dobbst@outlook.com (T.D.); 2 Clinical Neurological Sciences, London Health Sciences Centre, University of Western Ontario, London, ON N6A 5A5, Canada; E-Mails: patoriccio@gmail.com (P.R.); vladimir.hachinski@lhsc.on.ca (V.C.H.)

**Keywords:** subcortical ischemia, axonal degeneration, inflammation, secondary damage, executive function, frontal cortex

## Abstract

Subcortical ischemic strokes are among the leading causes of cognitive impairment. Selective atrophy of remote brain regions connected to the infarct is thought to contribute to deterioration of cognitive functions. The mechanisms underlying this secondary degenerative process are incompletely understood, but are thought to include inflammation. We induce ischemia by unilateral injection of endothelin-I into the rat dorsomedial thalamic nucleus, which has defined reciprocal connections to the frontal cortex. We use a comprehensive test battery to probe for changes in behaviour, including executive functions. After a four-week recovery period, brain sections are stained with markers for degeneration, microglia, astrocytes and myelin. Degenerative processes are localized within the stroke core and along the full thalamocortical projection, which does not translate into measurable behavioural deficits. Significant microglia recruitment, astrogliosis or myelin loss along the axonal projection or within the frontal cortex cannot be detected. These findings indicate that critical effects of stroke-induced axonal degeneration may only be measurable beyond a threshold of stroke severity and/or follow a different time course. Further investigations are needed to clarify the impact of inflammation accompanying axonal degeneration on delayed remote atrophy after stroke.

## 1. Introduction

Patients with ischemic stroke are at risk for developing vascular cognitive impairment; a profile mainly characterized by executive and attention deficits with a relatively intact memory function [1]. After a stroke, one in every five patients will become demented [2]. Subcortical ischemic strokes are among the leading causes of cognitive impairment [3], and although damage to strategic brain structures may partially explain cognitive impairment after stroke, other mechanisms, such as atrophy in selected remote regions, may play an equal or larger role. Exploring the relationship between cortical and subcortical infarcts and remote tissue degeneration is crucial to understand the underlying mechanisms leading to the progression of cognitive impairment.

There is clinical evidence showing that strokes located within the deep white matter can cause remote selective cortical and thalamic atrophy [4]. A diffusion tensor imaging (DTI) tractography study has shown remote focal cortical thinning in patients with subcortical infarcts [5]. Although the exact pathophysiological mechanism of cortical and subcortical grey matter atrophy after ischemic stroke is not known, the often described selective atrophy of remote regions connected to the stroke core points to a pathophysiological role for axonal degeneration [6]. One potential link between the degeneration of axonal pathways after stroke and remote neuronal damage is inflammation in the course of Wallerian degeneration [7,8]. Although inflammation can be beneficial in clearing degenerated cell material, it may also become harmful when not tightly regulated, and may promote secondary damage of healthy tissue [9,10].

Clinical studies have shown that acute subcortical ischemic strokes within the pyramidal tract trigger acute local microglia activation and a remote anterograde inflammatory process when measured at two weeks and at six months [11,12]. Furthermore, activated microglia were seen in PET studies after middle cerebral artery ischemic strokes in the contralateral hemisphere at 30 days [13] and in the ipsilateral and contralateral thalamus and pons after one [14] and five [15] months, respectively. In rat models of ischemic stroke, remote inflammatory infiltration [16] and neuronal damage [17] have also been observed in the ipsilateral thalamic nuclei after focal cortical cerebral ischemia. There is accumulating clinical and experimental evidence regarding acute [16] and chronic [18] post-stroke inflammatory processes taking place in remote areas. However, there is no evidence showing to what extent inflammatory mechanisms related to axonal degeneration may lead to cortical atrophy and subsequent cognitive impairment after a subcortical stroke.

To investigate the role of inflammation in remote pathological processes after ischemic stroke, we use a rat model of lacunar stroke in the dorsomedial thalamic nucleus (DMN). Lacunar stroke is of high clinical significance in this context since small vessel disease is one of the most common causes of cognitive impairment. Frequent locations for lacunar lesions include the thalamus, the striatum, and the internal capsule [3]. The thalamus is an essential node in various high-order cognitive circuits, and the DMN has strong neuronal connections with the prefrontal cortex [19,20,21]. In line with the observation of selective atrophy of regions connected to a stroke core, thalamic lesions affecting the DMN can cause frontal lobe impairment, such as executive and attention deficits [22]. The pathophysiological mechanism behind this clinical finding is incompletely understood, and likely involves reciprocal projections between the thalamus and the frontal cortex. As the ischemic lesion itself can often not exclusively explain the resulting cognitive impairment, we hypothesize that recruitment of microglia along the degenerating thalamocortical pathway may lead to inflammatory infiltration in the frontal cortex, thereby promoting secondary cortical neuronal degeneration.

**Figure 1:**
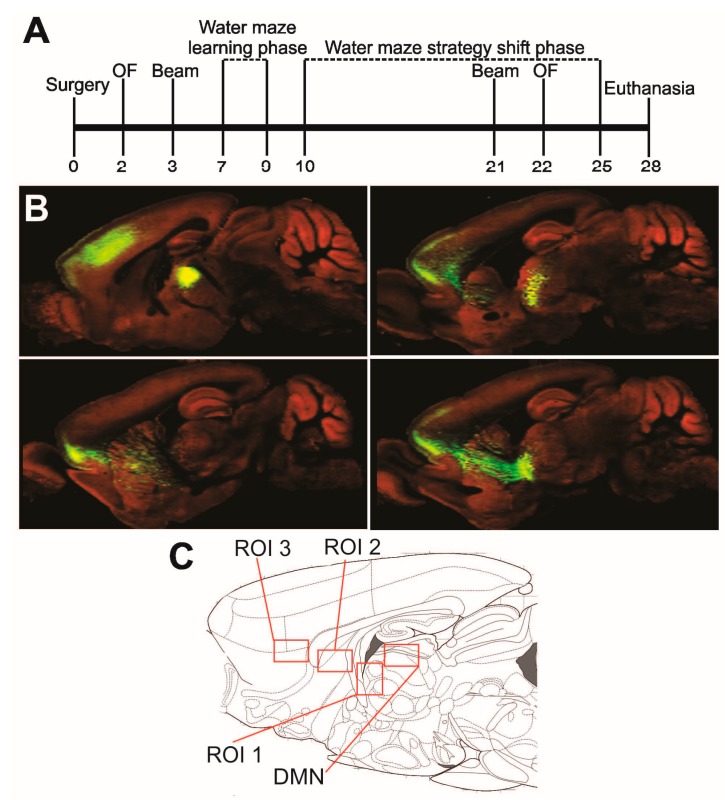
Experimental timeline and regions of interest. (**A**) Timeline demonstrates sequence of experimental procedures; (**B**) Dorsomedial thalamic nucleus (DMN) tracing in sagittal sections from lateral to medial reveals the connectivity between the mouse DMN and prefrontal cortex. Image credit: Allen Institute for Brain Science [23]; (**C**) The DMN and the three regions identified as regions of interest (ROIs) are marked in a schematic of a sagittal rat brain section at approximately 0.4 mm lateral to bregma (modified from Paxinos and Watson, 1998). ROI 1 = Periventricular white matter. ROI 2 = Striatal region of thalamocortical axon passage. ROI 3 = Deep layers of the frontal (pre-/infralimbic) cortex.

To assess this, we describe a comprehensive behavioural characterization of a rat model of defined DMN stroke, including assessment of executive function, over a post-stroke period of 28 days (Figure 1A). The reciprocal connectivity of the rat DMN and prefrontal cortex has been characterized in great detail anatomically using combined anterograde and retrograde tracing [20]. The Allen Institute of Brain Science’s Mouse Connectivity Brain Atlas shows this well-defined connectivity with outstanding clarity in all three dimensions [23] (Figure 1B). To establish what pathological changes have occurred along the thalamocortical pathway at four weeks post-stroke, we characterize the extent of degeneration, inflammation, astrogliosis and myelin content along the well-defined thalamocortical projection that originates in the DMN (Figure 1C) histologically in comparison with control animals.

## 2. Results

### 2.1. Open Field Activity and Anxiety-Related Behaviour

**Figure 2:**
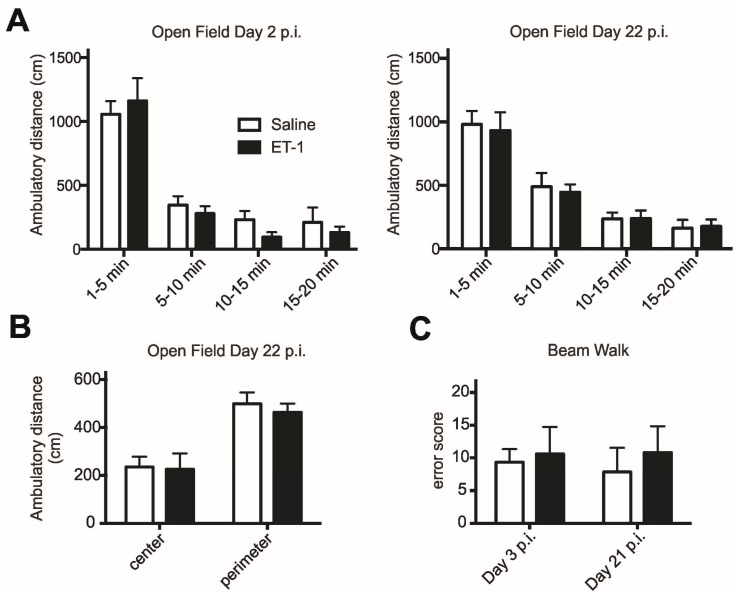
Behavioural assessments of activity, anxiety and motor control. (**A**) Activity in the open field assessed on day 2 and day 22 post injection (p.i.) does not differ statistically between experimental groups or time points (Two-way ANOVA); (**B**) On day 22 p.i., both animal groups are equally (Mann Whitney) more active in the perimeter zone than in the centre zone of the open field; and (**C**) Motor performance assessed while crossing an elevated beam is not significantly different between the animal groups at 3 days p.i. or at 21 days p.i. (Mann Whitney).

Spontaneous activity in the open field, measured by ambulatory time and distance (Figure 2A), as well as by the number of rears (not shown), did not differ between the two groups at either time point To detect a potential change in anxiety-related behaviour between animal groups or over the post-injection (p.i.) period, the time and distance that animals spent in the center zone of the open field *versus* the perimeter zone was plotted (Figure 2B). Comparisons showed that all animals behaved similarly at both time points measured, indicating no detectable change in anxiety-related behaviour in the open field.

### 2.2. Beam Walk Motor Performance

Motor control was assessed while animals were crossing an elevated narrow beam. Animals injected with ET-1 performed similar to control animals, and slips were due to missteps on either side of the body in both groups. Error scores did not differ between the first and second assessment post injection (Figure 2C).

**Figure 3:**
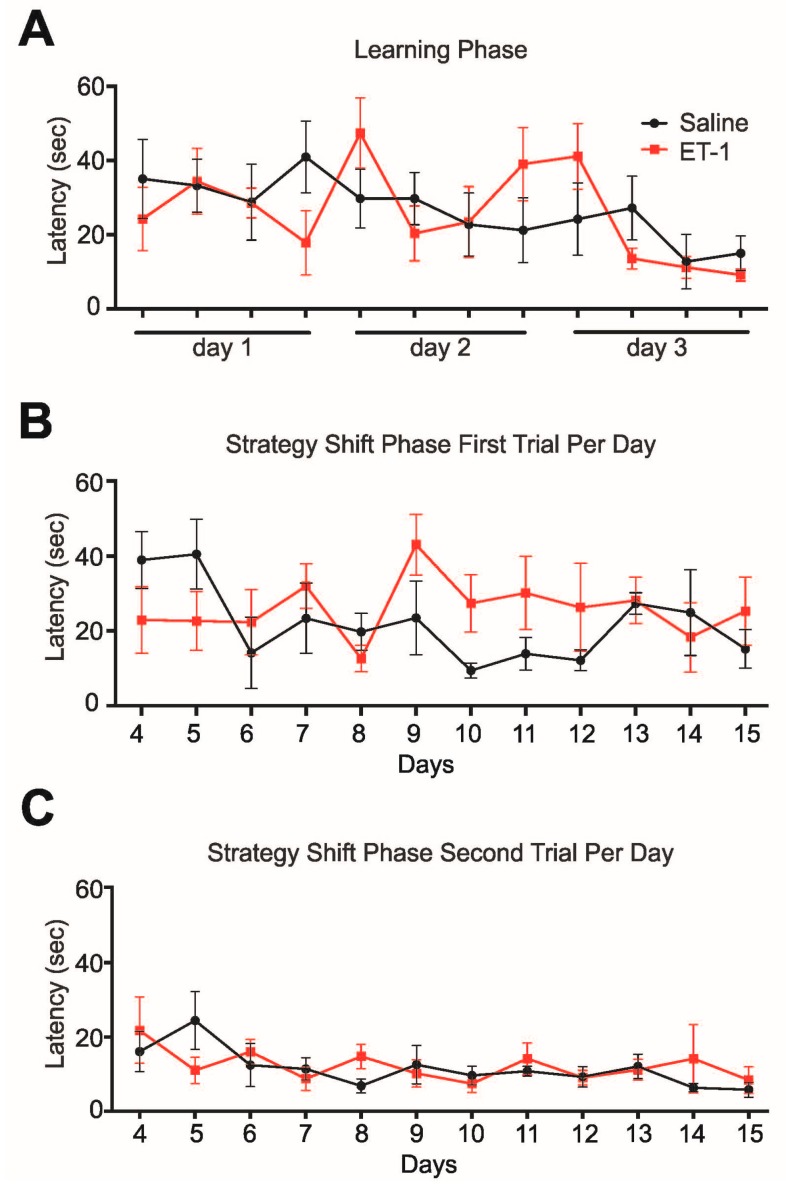
Assessments of executive function in the Morris Water Maze. (**A**) Both experimental groups learn to find the fixed hidden platform equally well across 12 learning trials, starting on day 7 p.i.; (**B**) Performance in the first trial of each day’s strategy shift protocol, when the platform was located in a new position, indicates no significant difference in performance between experimental groups; and (**C**) Performance on the second trial per day indicates no difference between the groups in the ability to recall the platform location from 30 s earlier.

### 2.3. Task Learning, Working Memory and Strategy Shift Testing in the Morris Water Maze

During the initial learning phase of the water maze of 12 trials across three days, both ET-1 injected and saline injected animals found the hidden platform within similar latencies. The day-to-day memory retention tended to be slightly better in control animals compared to ET-1 injected animals. Both animal groups learned to find the hidden platform on average within less than 15 s by the end of the 12-trial learning phase (Figure 3A). Starting on day 10 post-surgery, the location of the hidden platform was semi-randomly changed for the first out of 4 trials each day over a 12-day period. These trials were used to assess mental flexibility/strategy shift learning. Comparing the latencies to find the hidden platform at its ever-new location across 12 days, we observed a trend for ET-1 injected rats to take on average longer to achieve the task, especially on days 9 through 12 (Figure 3B). High inter-animal variability and limited animal numbers may have prevented this trend from reaching statistical significance across all time points. In the remaining three trials of each day (trials 2–4), the platform location remained the same as in the first trial of the respective day. These trials, with inter-trial intervals of only 30 s, were used as an indicator of the animals’ working memory. Both experimental groups performed similarly in this working memory assessment across the 12-day period, with improving latencies and declining variability across trials 2 to 4 on each day (trial 2 shown in Figure 3C). In summary, ET-1 injection did not result in changes in task learning, working memory, or strategy shift learning as measured with this water maze protocol.

### 2.4. Extent of Cellular Degeneration in the Thalamus

**Figure 4:**
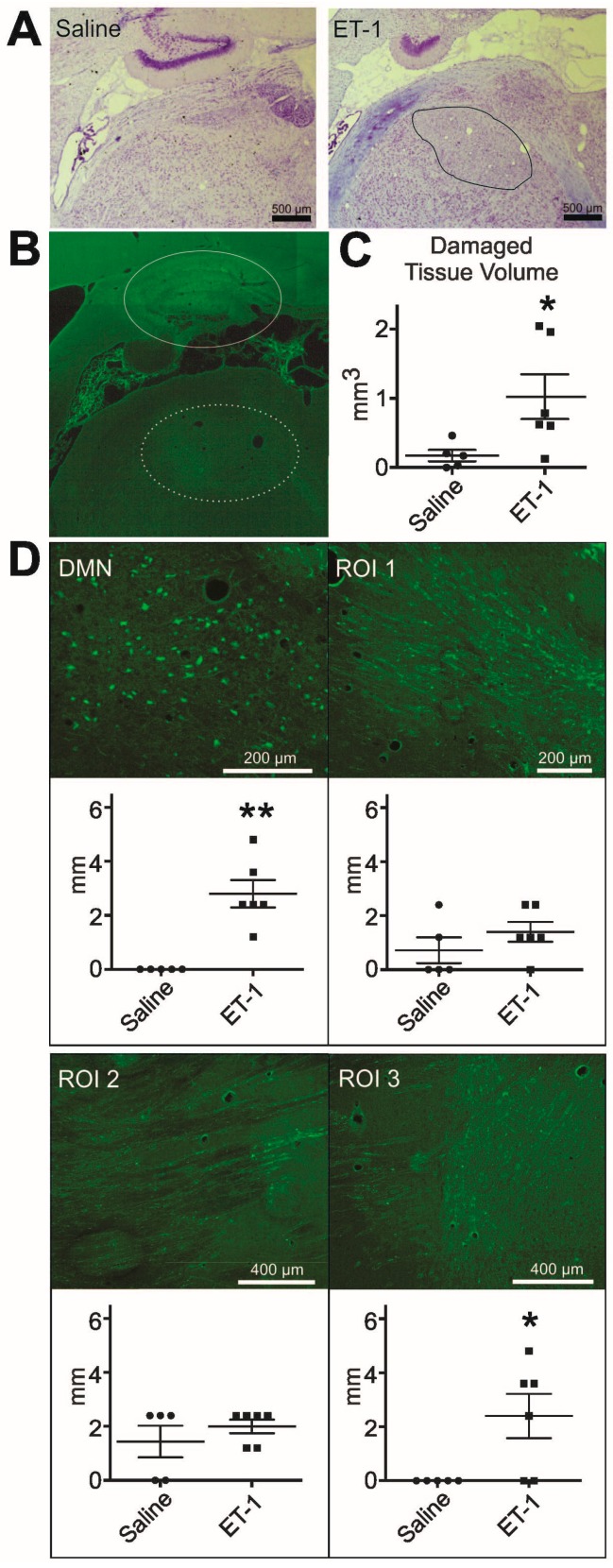
Degeneration in the injected region and along the thalamocortical pathway. (**A**) Thionine staining shows intact control DMN and tissue damage (delineated in black) in the DMN of an ET-1 injected brain; (**B**) FluoroJade B (FJB) positive signal in an image collage created from a sagittal section marks the region of ET-1 injection in the dorsal medial thalamic nucleus (DMN, dashed oval). The hippocampal formation is visible dorsal to the injection site (solid oval), rostral is to the left; (**C**) The area of damaged tissue in the injected region, as defined by FJB positive cells, is significantly greater in ET-1 injected brains than in their saline injected counterparts (Mann Whitney, * *p* = 0.03); and (**D**) Representative images show FJB positive signal in the DMN and the respective ROIs along the thalamocortical pathway. The lateral extent of FJB positive cells is significantly larger in the DMN (Mann-Whitney, ** *p* = 0.005) and the frontal cortex (ROI 3, Mann Whiteney, * *p* = 0.04) of ET-1 injected animals compared to saline injected rats.

Thionine staining of the infarcted region confirmed tissue damage consistent with ischemia in the DMN (Figure 4A). To estimate the volume of damaged tissue after ET-1 or saline injection, respectively, the area of FJB positive signal in the injected region (Figure 4B) was measured in consecutive sagittal sections. FJB is used here because it is sensitive enough to document ongoing degeneration as a result of mechanical damage following saline injection in control animals. The calculated total volume of FJB positive tissue in the thalamus was significantly higher in animals that had received an injection of ET-1 (1.023 mm^3^ ± 0.32) than in saline injected animals (0.174 mm^3^ ± 0.08, *p* = 0.03, Figure 3C). FJB positive signal was present in the DMN in all eleven brains analyzed except for one control animal.

### 2.5. Degeneration Outside the Dorsomedial Thalamic Nucleus

All FJB stained brains were screened for FJB positive signal in the injected region (DMN, Figure 4B–D), and along the thalamocortical projection pathway (Figure 1B,C and Figure 4D). The lateral extent of FJB positive signal was noted for each region. In the periventricular (ROI 1, Figure 4D) and striatal region (ROI 2, Figure 4D), FJB positive cells and fibres were observed more consistently in ET-1 injected brains than in their saline injected counterparts. However, 40% and 60% of control brains showed some extent of FJB signal in the periventricular and striatal region, respectively. No significant difference between the groups could be detected in these regions of thalamocortical fibre passage. In deep layers of the frontal cortex identified as infralimbic, prelimbic and cingulate cortex (ROI 3, Figure 4D), in close proximity to white matter, FJB positive signal was noted in four out of six ET-1 injected brains, and in none of the control brains (ET-1: 2.4 mm ± 0.82, saline: 0 mm ± 0, *p* = 0.04, ROI 3, Figure 4D). The positive signal was consistent with damaged fibres, and no FJB positive cell bodies could be identified (ROI 3, Figure 4D). These findings indicate that ET-1 injection into the DMN resulted in remote degenerative processes in the deep layers of the frontal cortex. Similar FJB signal was not consistently observed in the medial regions of the contralateral hemisphere.

Due to the injection route through the hippocampus, degeneration of sensitive hippocampal neurons could not be avoided. FJB-positive neurons were consistently present in several hippocampal regions, in some cases including the contralateral side as well. This bystander damage due to the mechanical injury was observed in four out of five control animals and in all six ET-1 injected brains. Cortical regions where the Hamilton needle passed through did not show FJB positive signal, yet FJB-positive axons could be identified in the corpus callosum in 73% of animals. Degenerating callosal fibres were in one case present independent of hippocampal damage.

**Figure 5:**
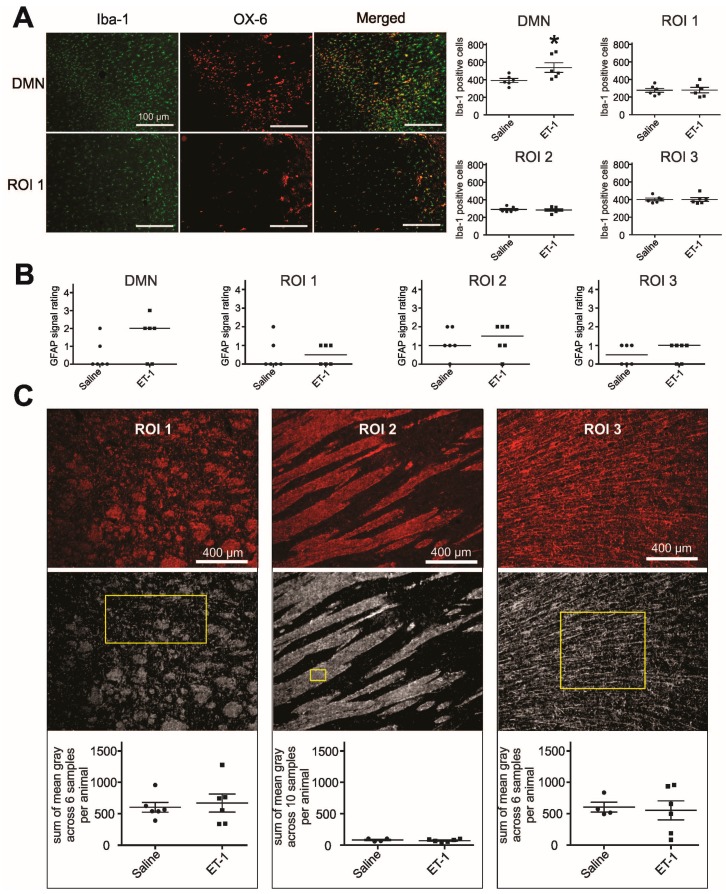
Inflammatory cells, astroglia and myelin content along the thalamocortical pathway. (**A**) Representative images taken from the DMN and the periventricular white matter (ROI 1) show Iba-1 and OX-6 positive cells. Quantification of Iba-1 positive cells across all regions analyzed shows that significantly more microglia are present in the DMN in ET-1 injected brains compared to saline injected brains (* *p* = 0.03); (**B**) Subjective rating of the extent of GFAP positive cells indicates that astroglial activation is similar across the ROIs and between the experimental groups; and (**C**) Images of sagittal sections immunostained for MBP (**top row**) were thresholded for densitometric analysis in ImageJ (**middle row**). Yellow boxes indicate size and shape of sampled area for each ROI. MBP density does not differ significantly between the groups in any ROI.

### 2.6. Quantification of Microglia and Astroglia

Degenerative processes are frequently accompanied by some response from microglia cells in the course of local inflammation. As inflammation may, ambivalently, play a role in clearing or in propagating tissue damage, characterizing the immune response surrounding degenerating thalamocortical fibres is pivotal for this study. To map the presence of highly activated, phagocytic microglia in our thalamocortical ROIs, tissue sections were stained for OX-6 (reacting with MHC-II, Figure 5A). The number of OX-6 positive cells, if any were present at all, was negligible in the periventricular, striatal and frontal lobe regions among all experimental animals. To allow quantification of recruited microglia that are not fully phagocytic, yet may be meaningful to the pathologic process, brain sections were stained with Iba-1 (Figure 5A). Iba-1 marks microglia cells irrespective of activation status. Comparison of the number of Iba-1 positive cells along the thalamocortical pathway did not yield a significant difference between the groups in any region (ROI 1–3, Figure 5A), including the frontal cortex (ROI 3). To validate our method of quantification of Iba-I positive cells, we also analyzed the injected thalamus. ET-1 injected brains were found to harbor significantly more microglia cells in the thalamus than saline injected brains (DMN, ET-1: 538.2 ± 54.54, saline: 391.3 ± 23.30, *p* = 0.03, Figure 5A).

Apart from microglia, astroglia likewise play an important part in the injured brain, and in the context of inflammatory processes. To assess the potential participation of astroglia in the process of remote degeneration after ischemia, we screened ROIs for the presence of activated astroglia cells. Such GFAP positive cells were present to similar extents in ET-1 compared to saline injected rat brains (Figure 5B), consistent with the number of Iba-1 positive microglia. In summary, no difference in the local response of microglia and astroglia could be detected along the thalamocortical pathway between ET-1 and saline injected brains.

### 2.7. Myelin Quantification along the Thalamocortical Pathway

To detect potential degradation of myelin, which may accompany the degeneration of fibre tracts, and which may influence the course of local inflammation, we quantified myelin basic protein (MBP) signal by densitometry in all regions of interest along the thalamocortical pathway (Figure 5C). Two saline-injected animals had to be excluded from this analysis in ROI 2 and ROI 3 due to insufficient tissue quality. Results did not indicate any trend for a change in myelin content between ET-1 and saline injected animals.

## 3. Discussion

White matter pathology following stroke has gained growing attention [12,24,25]. However, how degenerating axons can impact distant connected target regions is not well understood [17]. Hypotheses include the loss of inhibitory synaptic input to target neurons [17,26], excitotoxicity [27], and increased amyloid production [28]. A consistent finding in rat models of severe stroke is the presence of inflammatory markers in the connected region at early time points [16], often accompanied by neuronal degeneration. Since clinical evidence indicates that inflammatory responses within damaged white matter tracts can be prolonged [12,15], we hypothesize that this Wallerian degeneration of affected subcortical axons may sustain and further promote inflammation in distant cortical projection regions, leading to delayed atrophy. This hypothesis has to our knowledge not been addressed by previous studies, which often concentrate on acute effects and mostly rely on models of severe stroke with cortical involvement [24,29,30]. Experimentally inducing unilateral lacunar stroke in the DMN is a clinically relevant model to study remote effects of subcortical lacunar strokes, which in many cases lead to cognitive deterioration and delayed cortical atrophy in human subjects. Identifying the underlying mechanisms and the window of opportunity for treatment may open the door to preventing such delayed effects of stroke.

Using this animal model of DMN stroke, we observed degeneration of thalamocortical fibres along their full projection at four weeks post-stroke. Targeting the thalamocortical pathway offers the combined advantage of a (clinically relevant) subcortical stroke model and a defined, relatively long projection outside a major white matter tract. One limitation of targeting a subcortical area is undesired tissue damage by needle insertion through the cortex and hippocampus. Hippocampal degeneration was similar between saline and ET-1 injected animals, and would therefore be unlikely to contribute to group differences. However, it may make it more challenging to detect a behavioural effect of the lesion if control animals share some pathology with ET-1 injected rats. The observed lack of behavioural changes, particularly in assessments of executive function, can additionally be explained by the small size of the ischemic core [31]. Limiting the infarct size was considered necessary to produce a well-defined stroke, and unilateral stroke allowed for screening for distant changes in the contralateral hemisphere. However, a unilateral lesion of on average 1 mm^3^ volume may not be severe enough to produce deficits in executive function detectable with the methods and group sizes used herein. It may be that a bilateral and larger lesion [32], or multiple subcortical lacunar infarcts [33], are necessary to precipitate executive dysfunction. We can also not exclude that a unilateral DMN infarct combined with developing frontal cortex atrophy may lead to detectable behavioural deficits at later time points. Furthermore, when interpreting these results we should keep in mind that the rodent brain is generally found to be more resilient to injury than the human brain.

The four-week post-stroke recovery time, at which only axonal degeneration but no degeneration of neuronal cell bodies was observed in the frontal cortex, was primarily chosen to try to capture degenerative and inflammatory processes along the thalamocortical pathway. As the rat metabolism is higher than a human’s, delayed atrophy may occur sooner after the stroke than observed in patients [5]. Further investigation into the exact time course of secondary frontal cortex degeneration is needed to validate this model. Alternatively, neurodegeneration in the cortical projection region may more readily develop if the brain is already challenged, or “primed” for an inflammatory reaction [34]. Such a multifactorial explanation of secondary remote degeneration may include an additive or synergistic role of comorbidities, such as pre-existing cardiovascular risk factors, or changes in amyloid metabolism.

At four weeks post-stroke, axonal degeneration along the thalamocortical pathway was not accompanied by a significant inflammatory response, astrogliosis or myelin loss. This is an unexpected result as Wallerian degeneration, whose hallmark is inflammation, is known to occur in axons that degenerate as a result of axotomy [7] or due to death of the neuronal cell body [35]. Investigations into the inflammatory environment of degenerating thalamocortical fibres at earlier and later time points may clarify the pathophysiological process. If inflammation proves to be involved, it will be critical to relate pro-inflammatory processes to cortical neurodegeneration chronologically. Apart from the time course of events, inflammatory processes are inherently complex, with both beneficial and harmful potential [10,36]. It will therefore be important to characterize the nature of the response, specifically with respect to pro- and anti-inflammatory microglia phenotypes [37,38], to eventually identify an effective and safe target for immune-modulating interventions.

## 4. Materials and Methods

### 4.1. Experimental Groups and Surgical Procedures

All procedures involving animals were approved by the Western University Animal Care and Use Committee. Twelve 6-months old male Wistar rats (Charles River Canada), weighing between 644 and 747 grams at the beginning of the experiment, were housed separately and kept at a 12 h light/dark cycle. Six animals received an injection of 1 μL saline containing 10 pmol endothelin-1 (ET-1, E7764, Sigma-Aldrich, Oakville, ON, Canada) to induce ischemia in the DMN. The remaining six animals received an injection of 1 μL saline into the same location to serve as controls. All animals were anesthetized with isoflurane, mounted into a stereotaxic frame (David Kopf Instruments, Tujunga, CA, USA), and received a subcutaneous (s.c.) injection of the analgesic buprenorphine (0.03 mg/kg Temgesic, RB Pharmaceuticals Ltd., Berkshire, UK). Animals were kept on a heated blanket throughout the surgery. Following a small craniotomy at 2.9 mm posterior bregma and at 0.7 mm left to the midline, a Hamilton syringe (32 gauge) loaded with either ET-1 solution or saline, was lowered 5.3 mm below the dura. One microlitre was injected into the DMN slowly over the course of 2 min. The craniotomy was gently covered with bone wax (Ethicon, San Angelo, TX, USA), the skin incision sutured and the animal allowed to recover under a heat lamp. Post-operatively, all animals received 2 mL of saline s.c. for hydration and 0.03 mL of the antibiotic Baytril (Bayer, Toronto, ON, Canada) intramuscularly. Experimenters were blinded to the identity of the animals until completion of behavioural and histological data collection. A timeline of the experimental procedures is shown in Figure 1A.

### 4.2. Behavioural Assessments

Open field (43.2 cm by 43.2 cm, Med Associates Inc., St. Albans, VT, USA) locomotor and rearing activity was quantified over the course of 20 min on day 2 and day 22 after thalamic injection using an automated system (Activity Monitor software, Med Associates Inc.). A comparison between activity in the centre zone and the perimeter zone within the first 5 min of open field assessment was used as an indicator for anxiety-related behaviour.

As a measure of motor control, including foot placement and balance, rats were videotaped while crossing an elevated (40 cm high) narrow (2.3 cm wide, 1 m long) beam on days 3 and 21 post injection (p.i.). During subsequent frame-by-frame video analysis on-screen, foot slips and falls were counted in three ladder crossings in each direction. Slips were rated with an error score of 1, falls were rated with a score of 2. Error scores were summed up for each individual animal.

The Morris Water Maze apparatus was used to assess the rats’ working memory and their ability to detect changes in learned rules and to shift search strategies accordingly. Starting on day 7 after thalamic injection, all animals were given four 60 s trials a day, five days a week for the following three weeks to find a submerged platform. During the inter-trial interval of 30 s, rats were allowed to stay on the platform and were subsequently re-introduced into a new quadrant of the maze, rotating through all four quadrants in sequence for each trial. The platform location was fixed during the first three days (initial learning phase), and was moved to a different quadrant of the maze each day thereafter (strategy shift phase). No platform location was used more than once. Performance data was recorded using AnyMaze software (Stoelting, Wood Dale, IL, USA).

### 4.3. Perfusion and Tissue Preparation

Four weeks following thalamic injections, animals were euthanized with an overdose of pentobarbital (Pentobarbital Sodique, Ceva Santé Animale, ON, Canada) and perfused with heparinized saline followed by 4% paraformaldehyde. Brains were harvested, postfixed for 24 h in 4% paraformaldehyde, transferred to a 30% sucrose solution and frozen on dry ice. Sagittal cryosections of 20 μm thickness were mounted on superfrost slides (Fisher Scientific, Toronto, ON, Canada) in staggered fashion, yielding five tissue series throughout the left hemisphere and beyond with a 200 μm interval between neighbouring sections on each slide.

### 4.4. Thionine Staining

Slides were rehydrated in descending concentrations of ethanol, exposed to 0.5% Thionine for 40 s, washed in ddH_2_0, rehydrated in ascending concentrations of ethanol, immersed in xylene and coverslipped using Depex mounting medium (Electron Microscopy Sciences, VWR Canada, Mississauga, ON, Canada).

### 4.5. Fluoro Jade B Staining

Tissue sections were dehydrated at 37 °C for 1 h, then immersed in a 1% NaOH/80% ethanol solution for 5 min. Rehydration was achieved through 3 min immersions in decreasing concentrations of ethanol followed by 3 × 1 min double-distilled (dd) water. In a dark environment, slides were then incubated for 15 min in 0.06 M KMnO_4_, rinsed 3 × 1 min in ddH_2_O and subsequently incubated in 0.0004% Fluoro Jade B solution (FJB, Millipore, Temecula, CA, USA) for 20 min. Following 3 × 1 min rinses in dd water, slides were dehydrated at 37 °C for 25 min, immersed in xylene for 1 min and coverslipped.

### 4.6. Immunohistochemistry

Tissue slides were dehydrated at 37 °C for 1 h, then rinsed repeatedly in 0.01 M phosphate buffered saline (PBS). Except for Iba-I, unspecific antibody binding was blocked in 10% serum specific for the secondary antibody. Individual series of tissue sections were incubated with the following primary antibodies overnight at 4 °C: Rabbit anti-Iba-I to label microglia (1:1000, Wako Chemicals, Richmond, VA, USA), mouse anti-OX-6 to label MHC-II positive immune cells (1:100, BD Pharmingen, Mississauga, ON, Canada), mouse anti-GFAP to label astrocytes (1:100, Sigma), rabbit anti-MBP to label myelinated fibres (1:200, Abcam, Toronto, ON, Canada). Slides were then rinsed 3 × 10 min in PBS and incubated with secondary antibody (AF594 donkey anti-rabbit 1:100, Life Technologies Inc., Burlington, ON, Canada; FITC bovine anti-mouse 1:100, Santa Cruz, Dallas, TX, USA; for 2 h at room temperature. After 3 washes in PBS, slides were cover-slipped using Fluoroshield (Sigma) and stored at −20 °C.

### 4.7. Cellular Degeneration

All FJB stained tissue sections with positive signal in the thalamus were imaged at 10× with a Leica DM IRE2 microscope (Leica Microsystems Inc., Concord, ON, Canada), and the entire area of positive signal in the injected region was quantified using ImageJ 1.45 s software (Wayne Rasband, National Institute of Health, Bethesda, MD, USA). The full three-dimensional thalamic lesion extent was calculated from this data for each animal. In addition, all brain sections of the left hemisphere stained with FJB were screened throughout for positive signal, with focus on the DMN and its thalamocortical projections across the striatum into the prefrontal cortex. The number of consecutive slides containing FJB positive cells was noted together with the location of signal. Based on this analysis, regions of interest (ROIs) for all subsequent histological analyses were determined as follows: DMN, proximal thalamocortical axons with projections in the vicinity of the lateral ventricle (ROI 1), striatal regions of passage (ROI 2) and deep layers of the frontal cortex (ROI 3, Figure 1C). These regions were mostly limited to tissue sections between 0.3 and 1.5 mm lateral to midline, thus, further histological analyses were performed in these sections.

### 4.8. Quantification of Microglia and Astroglia

Iba-I positive cells were imaged at 10× in two non-neighbouring sections on two consecutive slides in ROIs. All images were processed in the same manner using ImageJ to allow for accurate automated cell counts using ImageJ’s particle analyzer. The sum of Iba-1 positive cells in four images per animal was used for statistical comparisons.

All ROIs were screened for OX-6 and GFAP positive cells. Baseline GFAP levels (non-activated cell state) were not detected with our immunohistochemical protocol, which allowed qualitative rating of the extent of GFAP positive signal (activated cells only) on an ordinal scale from 0 to 3 in each ROI.

### 4.9. Myelin Quantification

Images of MBP positive signal in the periventricular, striatal and frontal lobe regions were taken at 10x, and processed in ImageJ in the same manner across all images to optimize densitometric analysis. Densitometry of MBP positive signal was performed in three large sample areas per image in ROI 1 and 3, and in five small sample areas per image in ROI 2. Sample windows within the same ROI were of identical size and shape. The sums of density values for each animal were compared statistically.

### 4.10. Data Analysis

Histological data and outcome measures for anxiety and motor performance were compared using a Mann Whitney Test, water maze performance and open field behaviour were analyzed using a two-way ANOVA. A *p* value of ≤0.05 was considered significant. The group median is indicated for ordinal data (GFAP ratings), and the mean and SEM are indicated for nominal data. Group mean ± SEM are stated in the text.
